# Chloroplast genome characteristics and phylogenetic analysis of *Trevesia palmata* (Roxburgh ex Lindley) Visiani (Araliaceae)

**DOI:** 10.1080/23802359.2026.2657101

**Published:** 2026-04-15

**Authors:** Simin Chen, Chengming Wang, Yang Zhou, Yuanhui Chen, Siyu Chen, Ruihong Wang

**Affiliations:** College of Life Sciences and Medicine, Zhejiang Sci-Tech University, Hangzhou, China

**Keywords:** *Trevesia palmata*, Araliaceae, chloroplast genome, phylogenetic analysis

## Abstract

*Trevesia palmata* (Roxburgh ex Lindley) Visiani 1842 is a distinctive and visually striking plant species belonging to the family Araliaceae. Commonly known as the ‘Snowflake Aralia’ or ‘Spider Leaf Tree’, it is native to the subtropical and tropical regions of Southeast Asia. This study exhibits a complete chloroplast genome of *T. palmata*, alongside a phylogenetic analysis involving 23 additional species from Araliaceae. The chloroplast genome has a length of 156,541 bp and an overall GC content of 37.9%, which is structurally composed of a large single-copy (LSC) region (86,460 bp), a small single-copy (SSC) region (18,119 bp), and a pair of inverted repeat (IRs) regions (25,981 bp). At the gene level, this chloroplast genome harbors a total of 133 genes, comprising of 88 protein-coding genes, 37 tRNA genes and 8 rRNA genes. The phylogram showed that *T. palmata* shares a close evolutionary affinity with *Brassaiopsis* and *Eleutherococcus.* The chloroplast genome annotated and displayed below will lay a favorable foundation for the species relationship identification and evolutionary research of *Trevesia.*

## Introduction

*Trevesia* is a small genus of tropical and subtropical plants with only eight accepted species by now (Plants of the World Online, https://powo.science.kew.org/), belonging to the family Araliaceae, with *Trevesia palmata* (Roxburgh ex Lindley) Visiani 1842 (Visiani 1842) being the most prominent and widely recognized. *Trevesia palmata* typically grows as a small tree or shrub, reaching heights of 3–6 m, it is native to the subtropical and tropical regions of Southeast Asia, including parts of India, Nepal, Bhutan, Myanmar, and southern China (Wu and Raven 2007). It is renowned for its large, palmate leaves that are deeply lobed, giving them a unique, almost star-like appearance. The leaves are glossy and leathery, often with serrated edges, and can grow up to 30–60 cm in diameter, making them a prominent feature of the plant. Due to its ornamental foliage, *T. palmata* is sometimes cultivated in gardens and parks as an exotic decorative plant. Its unique leaf structure and tropical appearance make it a favorite among plant enthusiasts and horticulturists (Manoharan et al. 2023). Additionally, the plant has been used in traditional medicine for treating stomachache, dyspepsia, high blood pressure and postpartum care (Angami et al. 2006; Lalfakzuala et al. 2007; Lamxay et al. 2011). Phytochemical studies have shown that *T. palmata* contains abundant oleanane-type saponins with antimicrobial and antiproliferative activities. (Tommasi et al. 2000; Kim B et al. 2018). Overall, *T. palmata* is a fascinating species that combines ecological significance with medicinal value. Although recent studies have provided complete or nearly complete plastid genomes and nuclear genomic data for the Asian Palmate group of Araliaceae (e.g. the core Asian Palmate clade, Panax genus) (Valcárcel and Wen 2019; Gallego-Narbón et al. 2022; Kang et al. 2023; Gallego-Narbón et al. 2025), there is currently almost no research on the genomics of *T. palmata*, which restricts the molecular mechanism study of the discovered pharmacological effects and its precise application in the medical field. In this study, we assembled the chloroplast (cp) genome of *T. palmata* and conducted phylogenetic analysis. Our research prioritized the analysis of chloroplast genome features by means of detecting repetitive sequences and reconstructing phylogenetic trees. Beyond providing critical cp genome datasets, this study is also intended to advance our understanding of evolutionary connections among species within the Araliaceae family—filling key gaps in current knowledge of the family’s phylogenetic framework.

## Materials and methods

**Figure 1.**
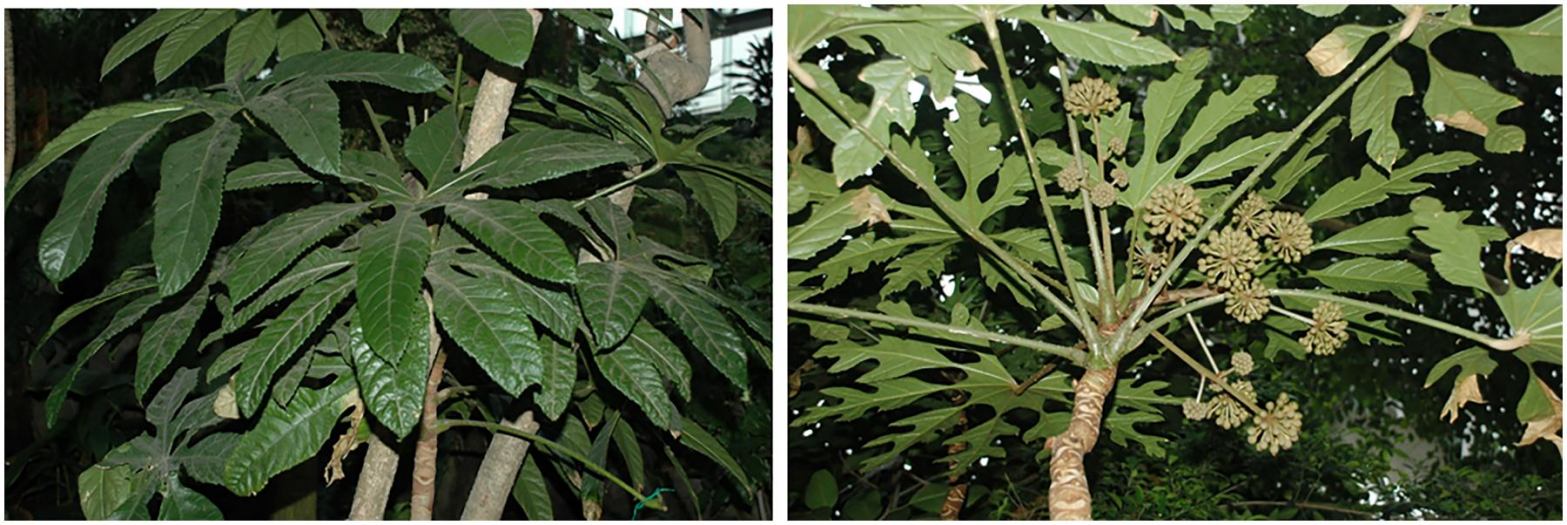
Reference image of *Trevesia palmata* by Difei Wu at Shanghai Botanical Garden and we have obtained permission to include the image in this article.

Fresh leaf samples of *T. palmata* were collected from Chenshan Botanical Garden (31.08236 N, 121.19382 E), deposited at Zhejiang Sci-Tech University identified by Zhechen Qi (Voucher No. HL00024, Hong Li, 19857120352@163.com) (Figure 1). Genomic DNA was meticulously extracted employing a modified CTAB protocol by using DNA Plantzol reagent (Invitrogen, Carlsbad, CA) to lyse plant tissues and remove polysaccharides and polyphenolic compounds (Liu J-L et al. 2018). Subsequent chloroform extraction separated contaminants from the aqueous phase containing nucleic acids. Finally DNA was precipitated with ethanol, yielding high-molecular-weight genomic DNA suitable for downstream applications. Libraries were constructed using a standard Illumina protocol involving DNA fragmentation, end repair, A-tailing, adapter ligation and PCR enrichment. The final libraries were sequenced using Illumina HiSeq 2500 platform with an average sequencing depth reaching 236× (Figure S1). Upon acquiring the raw sequencing data, Trimmomatic v0.39 (Bolger et al. 2014) was deployed to remove substandard reads and adapter sequences. The trimming was performed with standard parameters: ILLUMINACLIP was used to clip adapter sequences, followed by quality-based trimming with SLIDINGWINDOW (window size: 4 bp, required average quality: 15), and removal of leading/trailing bases with quality below 3. Reads shorter than 36 bp after trimming were discarded. We determined the per-base coverage depth across the *T. palmata* chloroplast genome with Bowtie 2 version 2.5.4 (Langmead and Salzberg 2012). Subsequently, we employed the GetOrganelle software in an iterative de novo assembly process (Jin JJ et al. 2020), involving read mapping and gap-filling, to reconstruct the entire plastome. The assembled plastomes were annotated using the GeSeq toolkit as implemented in Geneious Prime 2023.1. The genome map was plotted using the online tool Chloroplot (Zheng et al. 2020). The identification of cis-spliced and trans-spliced genes in the genome was conducted using CPGView (Liu C et al. 2012).

Phylogenetic reconstruction of *T. palmata* based on whole plastome data was performed with 23 closely related species from Araliaceae family downloaded from NCBI GenBank database, taking *Hydrocotyle vulgaris* and *Hydrocotyle sibthorpioides* as the outgroups (Table S1). The total of 24 plastome sequences was aligned using MAFFT V7 with default settings. Subsequently, a phylogenetic tree was constructed employing the maximum likelihood method through IQ-TREE V1.6.8 with the optimization model of TIM+F + R3 (Nguyen et al. 2015). MrBayes V3.2.7 was utilized to perform Bayesian inference phylogenetic tree construction under the best model of TPM1uf + I + G (Ronquist and Huelsenbeck 2003).

## Results

**Figure 2.**
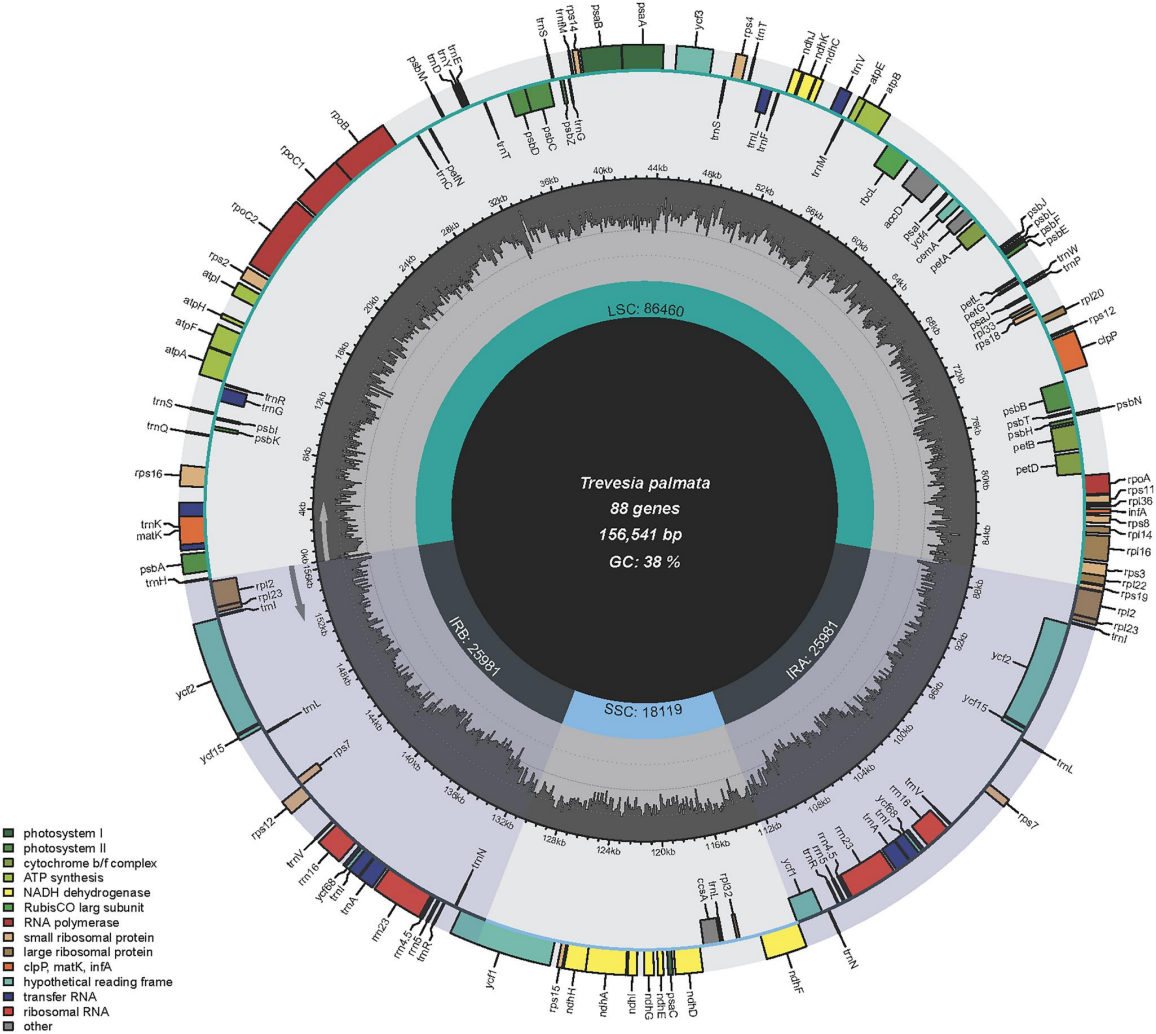
Chloroplast genome map of *Trevesia palmata. Notes:* Genes are depicted as differently sized and colored boxes on the outermost circle, with inner and outer boxes representing genes transcribed in clockwise and counter-clockwise directions. The middle circle illustrates changes in GC content at different positions, while the inner circle highlights the regions and lengths indicated by the tetrad structure (LSC, SSC, IRA, and IRB) in different colors.

The complete cp genome of *T. palmata* had a length of 156,541 bp, with an overall GC content of 37.9%. A conspicuous quadripartite structure observed in the assembled cp genome comprised a large single-copy (LSC: 86,460 bp) region, a small single-copy (SSC: 18,119 bp) region, and two inverted repeating regions (IRs: 25,981 bp) (Figure 2). The GC content differed among the four segments: the IR regions had the highest GC content at 43.03%, followed by the LSC region at 36.12%, while the SSC region had the lowest at 31.99%. Totally 133 functional genes were annotated in the *T. palmata* chloroplast genome, encompassing 88 protein-coding genes, 8 ribosomal RNA (rRNA) genes, 37 transfer RNA (tRNA) genes and 18 repeated genes. Of the 113 unique genes identified, eight protein-coding genes (*petB*, *petD*, *atpF*, *ndhA*, *rpl16*, *rpl2*, *rps16*, *rpoC1*) and six tRNA genes (*trnA-UGC*, *trnG-UCC*, *trnI-GAU*, *trnK-UUU*, *trnL-UAA*, *trnV-UAC*) were observed to have one intron each, while three genes (*clpP*, *rps12* and *ycf3*) were found to possess two introns, as demonstrated in Supplemental Figure S2.

**Figure 3.**
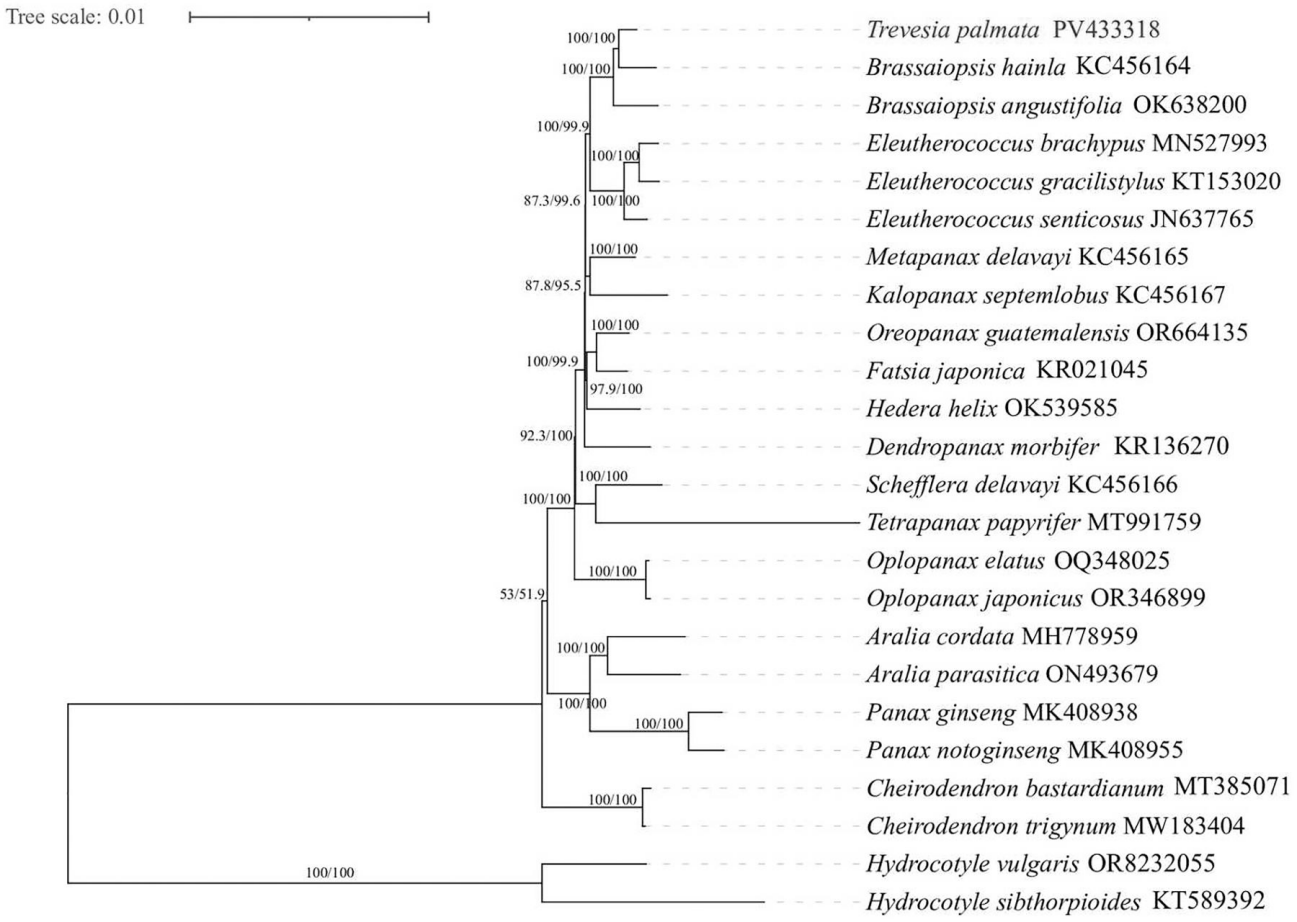
The phylogenetic tree based on the complete chloroplast genomes of 24 species of Araliaceae, with *Hydrocotyle, Cheirodendron, Panax* and *Aralia* as the outgroup. The number values at the nodes represent both the maximum likelihood bootstrap and the Bayesian inference posterior probability. The following details the sequence used to build the phylogenetic tree: the sequences of *Brassaiopsis hainla* KC456164 (Li et al. 2013), *Brassaiopsis angustifolia* OK638200 (Dong et al. 2022), *Eleutherococcus brachypus* MN527993 (Ou and Zhang 2020), *Eleutherococcus gracilistylus* KT153020 (Kim et al. 2016a,b), *Eleutherococcus senticosus* JN637765 (Yi et al. 2012), *Metapanax delavayi* KC456165 (Li et al. 2013), *Kalopanax septemlobus* KC456167 (Li et al. 2013), *Oreopanax guatemalensis* (OR664135), *Fatsia japonica* KR021045 (Chen et al. 2016)*, Hedera helix* (OK539585), *Dendropanax morbifer* KR136270 (Kim 2016a,b), *Schefflera delavayi* KC456166 (Li et al. 2013), *Tetrapanax papyrifer* (MT991759), *Oplopanax elatus* OQ348025 (Kang et al. 2023), *Oplopanax japonicus* OR346899 (Worth et al. 2024), *Aralia cordata* MH778959 (Kim CK and Kim 2019), *Aralia parasitica* (ON493679), *Panax ginseng* MK408938 (Ji et al. 2019), *Panax notoginseng* MK 408955 (Ji et al. 2019), *Cheirodendron bastardianum* MT385071 (Maurin 2020), *Cheirodendron trigynum* (MW183404), *Hydrocotyle vulgaris* OR8232055 (Luo et al. 2024) and *Hydrocotyle sibthorpioides* KT589392 (Ge et al. 2017).

Both Bayesian Inference (BI) and Maximum Likelihood (ML) approaches were utilized, yielding congruent topological results, as depicted in Figure 3. The phylogenetic analysis revealed that the majority of nodes were strongly supported with high bootstrap values and posterior probabilities. The outgroup species of *Hydrocotyle* occupied early-divergent positions within the phylogenetic tree, while the remaining 22 species from the Araliaceae family formed a monophyletic clade. Within this clade, several major lineages were resolved, although support values for some internal nodes varied. The genera *Cheirodendron*, *Panax*, and *Aralia* were each recovered as monophyletic with maximal support (100/100). Within the remaining taxa, the genus *Oplopanax* diverged first, followed by the *Schefflera*-*Tetrapanax* branch, with subsequent divergences of *Dendropanax*, *Hedera*, *Oreopanax*-*Fatsia* and *Metapanax-Kalopanax*. At the innermost position of this phylogenetic tree, *Trevesia palmata* formed a sister group with *Brassaiopsis hainla* and *Brassaiopsis angustifolia*, which in turn was sister to the genus *Eleutherococcus*.

## Discussion and conclusion

This topology demonstrates that leaf morphology (palmate vs. pinnate) is homoplastic and does not predict phylogenetic relationships among these genera. For instance, *Fatsia* with palmate leaves and *Hedera* and *Schefflera* with pinnate leaves belong to the same branch, while other palmate leaf genera (such as *Trevesia* and *Brassaiopsis*) are located in different positions. This strongly supports the conclusion of Wen et al. (2001) and Plunkett et al. (2004), that the leaf shape trait is independently evolved multiple times (convergent) within the Araliaceae family, and cannot be used as a reliable basis for classifying major group. When comparing the data results with similar studies, we found that the chloroplast genome sequence of *Trevesia palmata* in this study shares partial similarity and data overlap with the previously published *Trevesia palmata* sequences with accession numbers of ON881739, and OQ397539 on NCBI (Jin L et al. 2024). To highlight the distinctive characteristics of the sequence of this study contrasted with above two sequences, we will explain from the following two aspects. Firstly, the annotation information of sequence ON881739 is incomplete. Secondly, the essential difference between the *Trevesia palmata* sequence in this study and sequence OQ397539 lies in the different orientations of the small single-copy (SSC) region. Analysis results show that the orientation of the SSC region in PV433318 is consistent with that of other species within the genus *Trevesia* and species of the closest related genera. However, the orientation of the SSC region in sequence OQ397539 is inverted. This finding strongly indicates that the assembly result of OQ397539 may have an artificial orientation error, while the PV433318 submitted by us accurately represents the true evolutionary genomic structure of this species.

Therefore, in this study, we sequenced and characterized the complete chloroplast genome of *T. palmata*, revealing its canonical quadripartite and circular structure. Our analyses disclosed notable structural and sequence variations, including differences in genome size, gene content, and intron composition. Phylogenetic reconstruction based on chloroplast genome data robustly placed *T. palmata* as closely related to the genera *Brassaiopsis* and *Eleutherococcus*, providing new insights into the evolutionary relationships among their plastid genomes within Araliaceae. The release of *T. palmata* cp genome (accession number PV433318) not only provides a high-quality reference sequence with annotations but, more importantly, corrects a potential error in the existing database. This genomic resources offers valuable data for future studies on plastid genome evolution, comparative genomics, and for inferring phylogenetic relationships within this group when combined with additional evidence.

## Supplementary Material

Supplementary materials 20260306.docx

## Data Availability

The genome sequence data that support the findings of this study are openly available in GenBank of NCBI at (https://www.ncbi.nlm.nih.gov/) under the accession no. PV433318. The associated BioProject, SRA, and Bio-Sample numbers are PRJNA1247797, SRR33016301 and SAMN47830244 respectively.

## References

[CIT0001] Angami A, Gajurel PR, Rethy P, Singh B, Kalita SK. 2006. Status and potential of wild edible plants of Arunachal Pradesh. Indian J Tradit Knowl. 5(4):541–550.

[CIT0002] Bolger AM, Lohse M, Usadel B. 2014. Trimmomatic: a flexible trimmer for Illumina sequence data. Bioinformatics. 30(15):2114–2120. 10.1093/bioinformatics/btu170 24695404 PMC4103590

[CIT0003] Chen Q et al. 2016. The complete chloroplast genome sequence of *Fatsia japonica* (Apiales: Araliaceae) and the phylogenetic analysis. Mitochondrial DNA A DNA Mapp Seq Anal. 27(4):3050–3051. 10.3109/19401736.2015.1063129 26153743

[CIT0004] Dong Z et al. 2022. The complete plastid genome of the endangered shrub *Brassaiopsis angustifolia* (Araliaceae): comparative genetic and phylogenetic analysis. PLoS One. 17(6):e0269819. 10.1371/journal.pone.0269819 35771795 PMC9246242

[CIT0005] Gallego-Narbón A et al. 2025. Ancient polyploidization events influence the evolution of the ginseng family (Araliaceae). Front Plant Sci. 16:1595321. 10.3389/fpls.2025.1595321 40584867 PMC12202383

[CIT0006] Gallego-Narbón A, Wen J, Liu J, Valcárcel V. 2022. Hybridization and genome duplication for early evolutionary success in the Asian Palmate group of Araliaceae. J Syst Evol. 60(6):1303–1318. 10.1111/jse.12906

[CIT0007] Ge L et al. 2017. The complete chloroplast genome sequence of *Hydrocotyle sibthorpioides* (Apiales: Araliaceae). Mitochondrial DNA B Resour. 2(1):29–30. 10.1080/23802359.2016.1241676 33473705 PMC7800476

[CIT0008] Ji Y et al. 2019. Testing and using complete plastomes and ribosomal DNA sequences as the next generation DNA barcodes in *Panax* (Araliaceae). Mol Ecol Resour. 19(5):1333–1345. 10.1111/1755-0998.13050 31237984

[CIT0009] Jin JJ et al. 2020. GetOrganelle: a fast and versatile toolkit for accurate de novo assembly of organelle genomes. Genome Biol. 21(1):241. 10.1186/s13059-020-02154-5 32912315 PMC7488116

[CIT0010] Jin L et al. 2024. Stronger latitudinal phylogenetic patterns in woody angiosperm assemblages with higher dispersal abilities in China. J Biogeogr. 51(2):269–279. 10.1111/jbi.14746

[CIT0011] Kang J-S et al. 2023. Evolution of the Araliaceae family involved rapid diversification of the Asian Palmate group and *Hydrocotyle* specific mutational pressure. Sci Rep. 13(1):22325. 10.1038/s41598-023-49830-7 38102332 PMC10724125

[CIT0012] Kim B et al. 2018. Identification of novel compounds, oleanane and ursane-type triterpene glycosides from *Trevesia palmata*: their biocontrol activity against phytopathogenic fungi. Sci Rep. 8(1):14522. 10.1038/s41598-018-32956-4 30266953 PMC6162204

[CIT0013] Kim CK, Kim YK. 2019. The complete chloroplast genome of *Aralia cordata* (Apiales: Araliaceae). Mitochondrial DNA Part B. 4(1):211–212. 10.1080/23802359.2018.1546140

[CIT0014] Kim K et al. 2016a. The complete chloroplast genome of *Eleutherococcus gracilistylus* (WW Sm.) SY Hu (Araliaceae). Mitochondrial DNA A DNA Mapp Seq Anal. 27(5):3741–3742. 10.3109/19401736.2015.1079884 26358682

[CIT0015] Kim K, Lee SC, Yang TJ. 2016b. The complete chloroplast genome sequence of *Dendropanax morbifera* (Leveille). Mitochondrial DNA A DNA Mapp Seq Anal. 27(4):2923–2924. 10.3109/19401736.2015.1060442 26153746

[CIT0016] Lalfakzuala R, Lalramnghinglova H, Kayang H. 2007. Ethnobotanical usages of plants in Western Mizoram. Indian J Tradit Knowl. 6(3):486–493.

[CIT0017] Lamxay V, de Boer HJ, Bjork L. 2011. Traditions and plant use during pregnancy, childbirth and postpartum recovery by the Kry ethnic group in Lao PDR. J Ethnobiol Ethnomed. 7(1):1–16. 10.1186/1746-4269-7-14 21569234 PMC3120637

[CIT0018] Langmead B, Salzberg S. 2012. Fast gapped-read alignment with Bowtie 2. Nat Methods. 9(4):357–359. 10.1038/nmeth.1923 22388286 PMC3322381

[CIT0019] Li R et al. 2013. Complete sequencing of five Araliaceae chloroplast genomes and the phylogenetic implications. PLoS One. 8(10):e78568. 10.1371/journal.pone.0078568 24205264 PMC3799623

[CIT0020] Liu C et al. 2012. CpGAVAS, an integrated web server for the annotation, visualization, analysis, and GenBank submission of completely sequenced chloroplast genome sequences. BMC Genomics. 13(1):715. 10.1186/1471-2164-13-715 23256920 PMC3543216

[CIT0021] Liu J-L et al. 2018. The complete chloroplast genome of the Chinese Abelia *Lineae chinensis* (R.Br.) A. Braun & Vatke (Caprifoliaceae). Mitochondrial DNA Part B. 3(2):1081–1082. ** 10.1080/23802359.2018.1508386 33490560 PMC7800988

[CIT0022] Luo X et al. 2024. The complete chloroplast genome sequence of *Hydrocotyle vulgaris* L.(Araliaceae). Mitochondrial DNA B Resour. 9(5):647–651. 10.1080/23802359.2024.2349333 38770144 PMC11104692

[CIT0023] Manoharan AL et al. 2023. Efficacy of *Trevesia palmata* (Roxb. ex Lindl.) Vis. Extract on MG 63 cell lines and arthritis-induced animal models. J Ethnopharmacol. 300:115742. 10.1016/j.jep.2022.115742 36152784

[CIT0024] Maurin KJL. 2020. A dated phylogeny of the genus *Pennantia* (Pennantiaceae) based on whole chloroplast genome and nuclear ribosomal 18S–26S repeat region sequences. PhytoKeys. 155:15–32. 10.3897/phytokeys.155.53460 32863722 PMC7428460

[CIT0025] Nguyen LT, Schmidt HA, Von Haeseler A, Minh BQ. 2015. IQ-TREE: a fast and effective stochastic algorithm for estimating maximum-likelihood phylogenies. Mol Biol Evol. 32(1):268–274. 10.1093/molbev/msu300 25371430 PMC4271533

[CIT0026] Ou XB, Zhang DH. 2020. The complete chloroplast genome of *Acanthopanax brachypus* (Araliaceae), a famous medicinal plant in China. Mitochondrial DNA B Resour. 5(3):2709–2710. 10.1080/23802359.2020.1787888 33457915 PMC7781937

[CIT0027] Plunkett GM, Wen J, Lowry PP. 2004. Infrafamilial classifications and characters in Araliaceae: insights from the phylogenetic analysis of nuclear (ITS) and plastid (*trn*L-*trn*F) sequence data. Plant Syst Evol. 245(1-2):1–39. 10.1007/s00606-003-0101-3

[CIT0028] Ronquist F, Huelsenbeck JP. 2003. MrBayes 3: Bayesian phylogenetic inference under mixed models. Bioinformatics. 19(12):1572–1574. 10.1093/bioinformatics/btg180 12912839

[CIT0029] Tommasi ND et al. 2000. Antiproliferative triterpene saponins from *Trevesia palmata*. J Nat Prod. 63(3):308–314. 10757708 10.1021/np990231n

[CIT0030] Valcárcel V, Wen J. 2019. Chloroplast phylogenomic data support Eocene amphi-Pacific early radiation for the Asian Palmate core Araliaceae. J Syst Evol. 57(6):547–560. 10.1111/jse.12522

[CIT0031] Visiani R. 1842. Memorie della Reale Accademia delle Scienze di Torino, Serie 2. Mem R Accad Sci Torino 4: 262.

[CIT0032] Wen J, Plunkett GM, Mitchell AD. 2001. The evolution of Araliaceae: a phylogenetic analysis based on ITS sequences of nuclear ribosomal DNA. Syst Bot. 26(1):144–167. 10.2307/2666661

[CIT0033] Worth JRP et al. 2024. Chloroplast genome‐based genetic resources via genome skimming for the subalpine forests of Japan and adjacent regions. Ecol Evol. 14(7):e11584. 10.1002/ece3.11584 39026955 PMC11255381

[CIT0034] Wu ZY, & Raven PH. (eds.). 2007. *Trevesia palmata*. In Flora of China, Vol. 13 (Araliaceae), p. 456–457. Science Press, Beijing, and Missouri Botanical Garden Press.

[CIT0035] Yi D-K et al. 2012. The complete chloroplast DNA sequence of *Eleutherococcus senticosus* (Araliaceae); comparative evolutionary analyses with other three asterids. Mol Cells. 33(5):497–508. 10.1007/s10059-012-2281-6 22555800 PMC3887725

[CIT0036] Zheng SY, Poczai P, Hyvönen J, Tang J, Amiryousefi A. 2020. Chloroplot: an online program for the versatile plotting of organelle genomes. Front Genet. 11:576124. 10.3389/fgene.2020.576124 33101394 PMC7545089

