# Supplementary material for: Chloroplast genome characteristics and phylogenetic analysis of Trevesia palmata (Roxburgh ex Lindley) Visiani (Araliaceae)
Source: Mitochondrial DNA B Resour. 2026 Apr 15;11(5):619–24. doi: 10.1080/23802359.2026.2657101 (PMC13084832; doi:10.1080/23802359.2026.2657101)

Chloroplast genome characteristics and phylogenetic analysis of *Trevesia palmata* (Roxburgh ex Lindley) Visiani (Araliaceae)

Simin Chen, Chengming Wang, Yang Zhou, Yuanhui Chen, Siyu Chen, and Ruihong Wang*

College of Life Sciences and Medicine, Zhejiang Sci-Tech University, Hangzhou, China

*Corresponding authour: ruihong8977@zstu.edu.cn


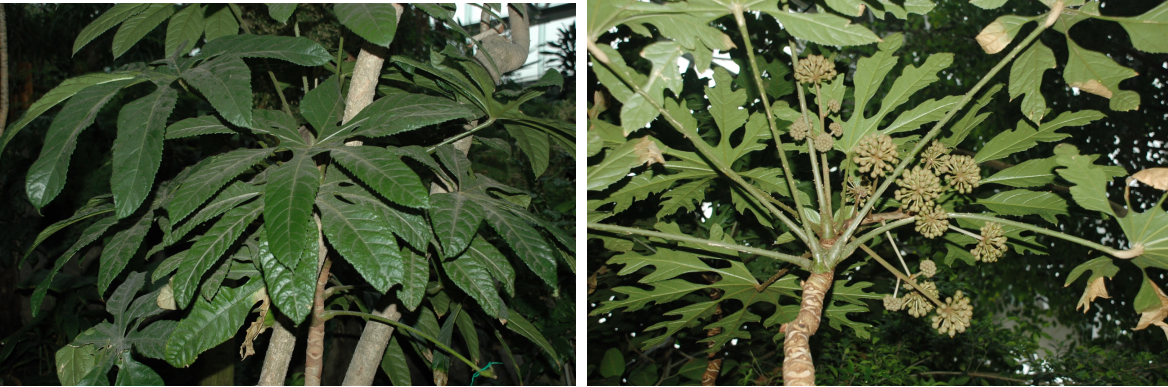


Mitochondrial DNA Part B: Resources

Online ISSN: 2380-2359

**Supplementary materials:**

Table S1. The scientific names and the NCBI number of chloroplast genomes for 24 species in family Araliaceae.

| Genus | Species | NCBI accession no. |
| --- | --- | --- |
| *Trevesia* | *Trevesia palmata* | PV433318 |
| *Brassaiopsis* | *Brassaiopsis hainla* | KC456164 |
| *Brassaiopsis* | *Brassaiopsis angustifolia* | OK638200 |
| *Eleutherococcus* | *Eleutherococcus brachypus* | MN527993 |
| *Eleutherococcus* | *Eleutherococcus gracilistylus* | KT153020 |
| *Eleutherococcus* | *Eleutherococcus senticosus* | JN637765 |
| *Metapanax* | *Metapanax delavayi* | KC456165 |
| *Kalopanax* | *Kalopanax septemlobus* | KC456167 |
| *Oreopanax* | *Oreopanax guatemalensis* | OR664135 |
| *Fatsia* | *Fatsia japonica* | KR021045 |
| *Hedra* | *Hedra helix* | OK539585 |
| *Dendropanax* | *Dendropanax morbifer* | KR136270 |
| *Schefflera* | *Schefflera delavayi* | KC456166 |
| *Tetrapanax* | *Tetrapanax papyrifer* | MT991759 |
| *Oplopanax* | *Oplopanax elatus* | OQ348025 |
| *Oplopanax* | *Oplopanax* japonicus | OR346899 |
| *Aralia* | *Aralia cordata* | MH778959 |
| *Aralia* | *Aralia* parasitica | ON493679 |
| *Panax* | *Panax ginsen* | MK408938 |
| *Panax* | *Panax notoginsene* | MK 408955 |
| *Cheirodendron* | *Cheirodendron* *bastardianum* | MT385071 |
| *Cheirodendron* | *Cheirodendron trigynum* | MW183404 |
| *Hydrocotyle* | *Hydrocotyle vulgaris* | OR8232055 |
| *Hydrocotyle* | *Hydrocotyle sibthorpioides* | KT589392 |


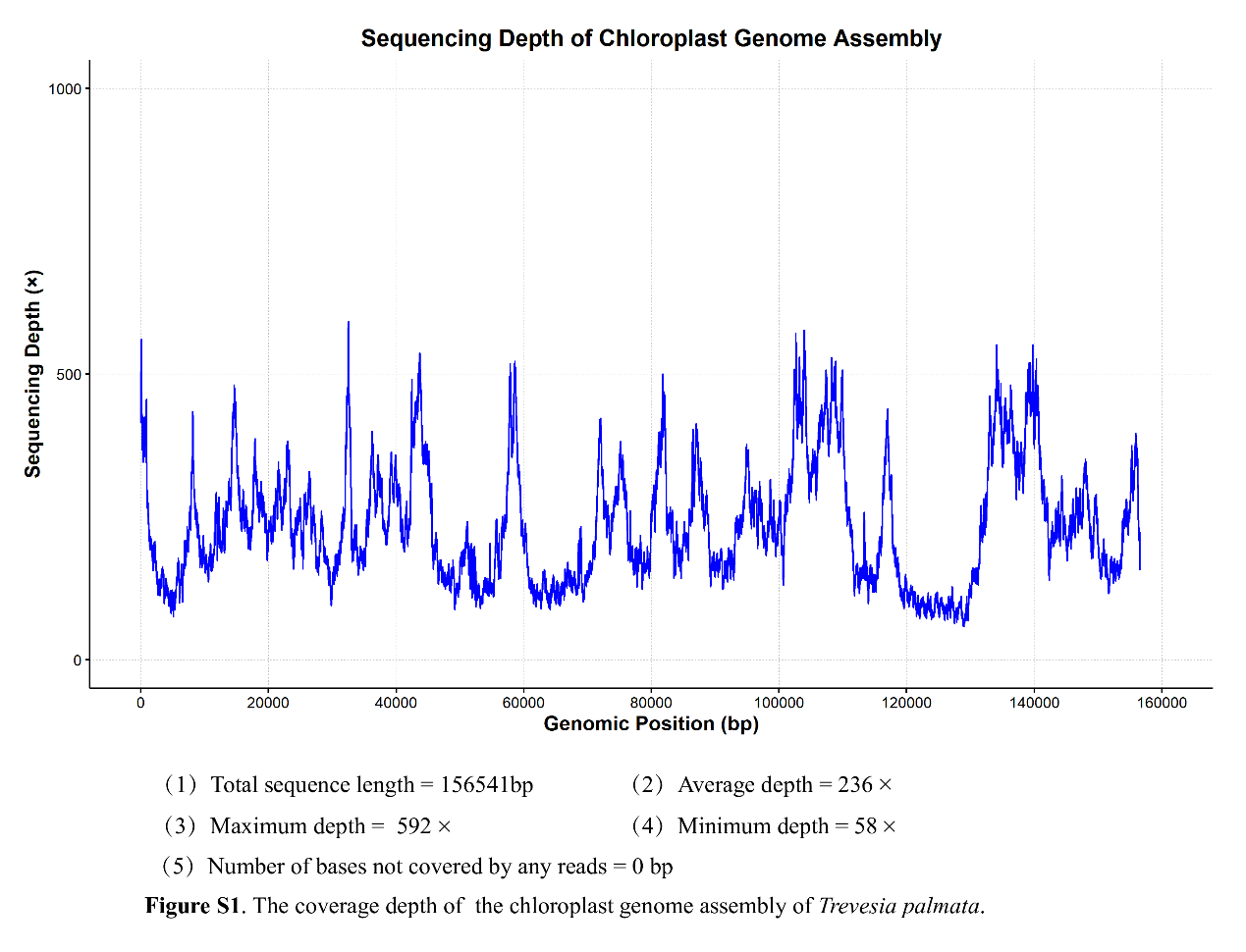


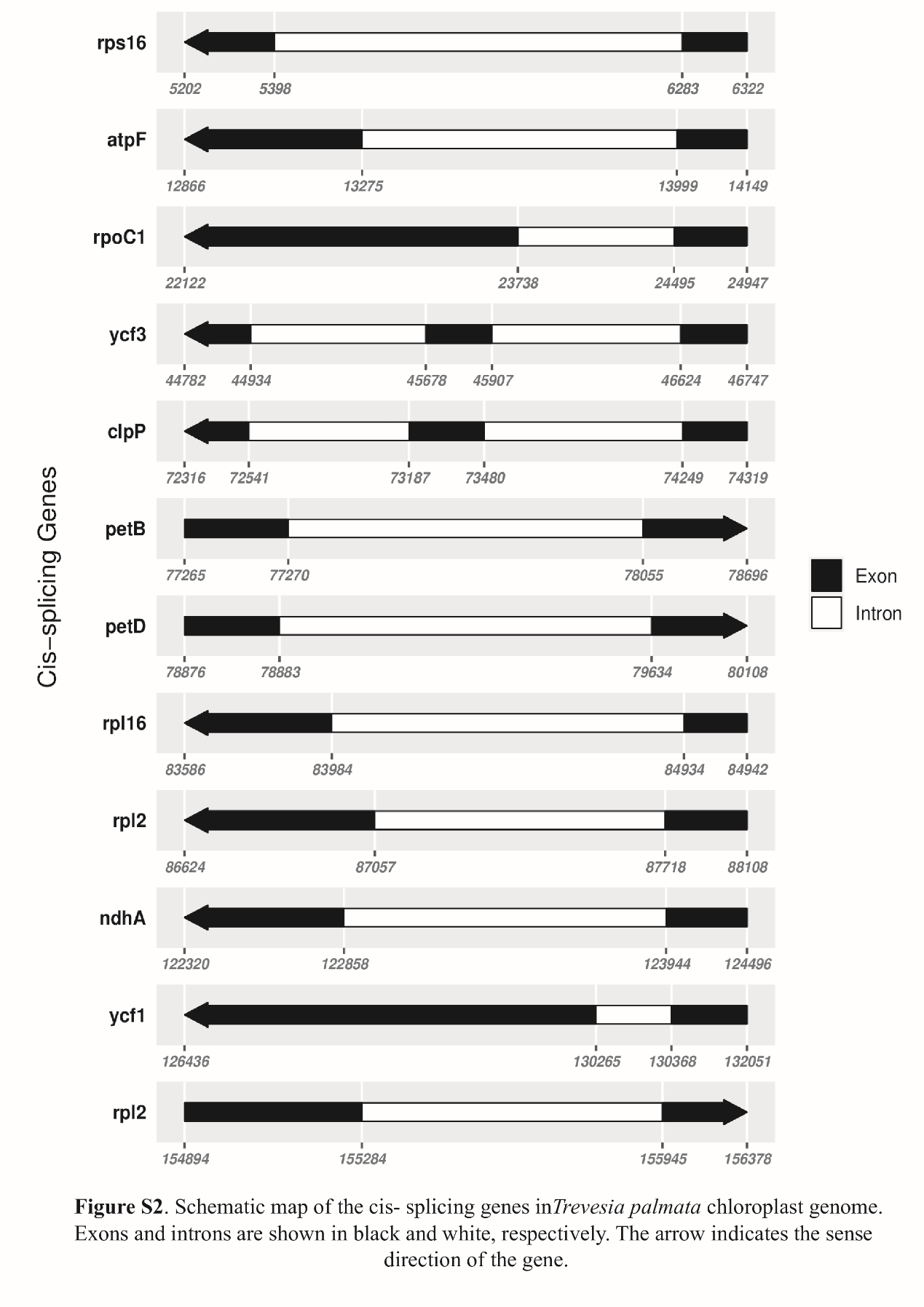


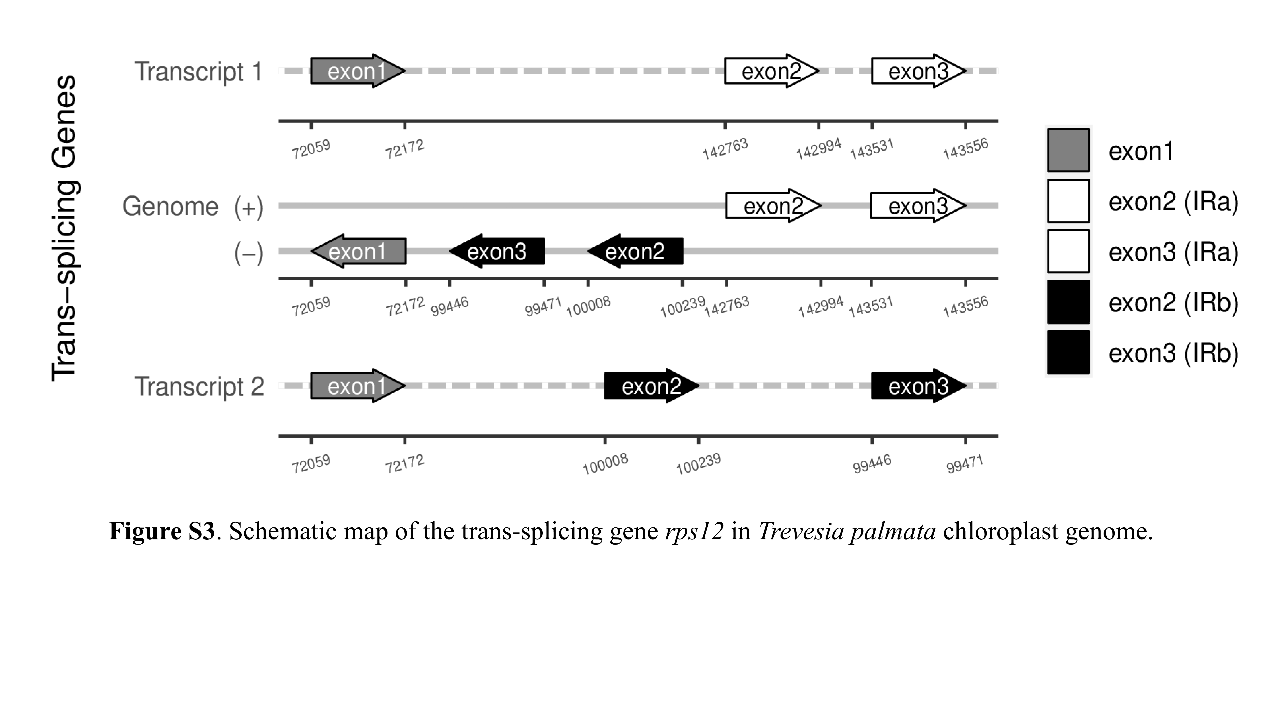

Supplement: Supplementary materials 20260306.docx [file TMDN_A_2657101_SM4772.docx]
